# Editorial: Biochar and nanoparticles as tools for enhancing plant resilience to biotic and abiotic stresses

**DOI:** 10.3389/fpls.2026.2003282

**Published:** 2026-10-05

**Authors:** Raul Antonio Sperotto, Halley Caixeta Oliveira, Zsuzsanna Kolbert, Yaosheng Wang

**Affiliations:** 1 Brazilian Agriculture Research Corporation (Embrapa Maize and Sorghum), Sete Lagoas, Brazil; 2 Laboratory of Biodiversity (LABIO), Department of Animal and Plant Biology, State University of Londrina, Londrina, Brazil; 3 Department of Plant Biology, University of Szeged, Szeged, Hungary; 4 Institute of Environment and Sustainable Development in Agriculture, Chinese Academy of Agricultural Sciences, Beijing, China

**Keywords:** biochar, nanoparticles, plant stress resilience, soil-plant interactions, sustainable agriculture

## 1 Introduction

Agricultural production is increasingly challenged by multiple abiotic and biotic stresses, including drought, salinity, soil contamination, nutrient limitations, and pests (Choudhary et al., 2025). Climate change is expected to further increase the frequency and severity of many of these constraints, reinforcing the need for strategies capable of improving plant resilience while reducing environmental impacts (Zhang et al., 2025). Within this context, biochar and nanoparticles have emerged as promising tools because they can act at multiple levels of the soil–plant system, influencing nutrient availability, water relations, redox homeostasis, stress signaling, and interactions with the surrounding environment (Bezzalla et al., 2026; Ghafoor et al., 2026; Sultan et al., 2026). This Research Topic “*Biochar and Nanoparticles as Tools for Enhancing Plant Resilience to Biotic and Abiotic Stresses*” brings together six contributions that illustrate a transition from proof-of-concept applications toward a more integrated understanding of how these materials influence plant performance under stress. Collectively, the articles emphasize that demonstrating increases in biomass or growth alone is not sufficient to establish the mechanisms underlying enhanced stress resilience, as suggested by Sperotto et al. (2026). A more robust understanding requires linking material properties with soil processes, plant physiology, metabolism, and molecular responses within an integrated soil–plant framework (**Figure 1**).

**Figure 1:**
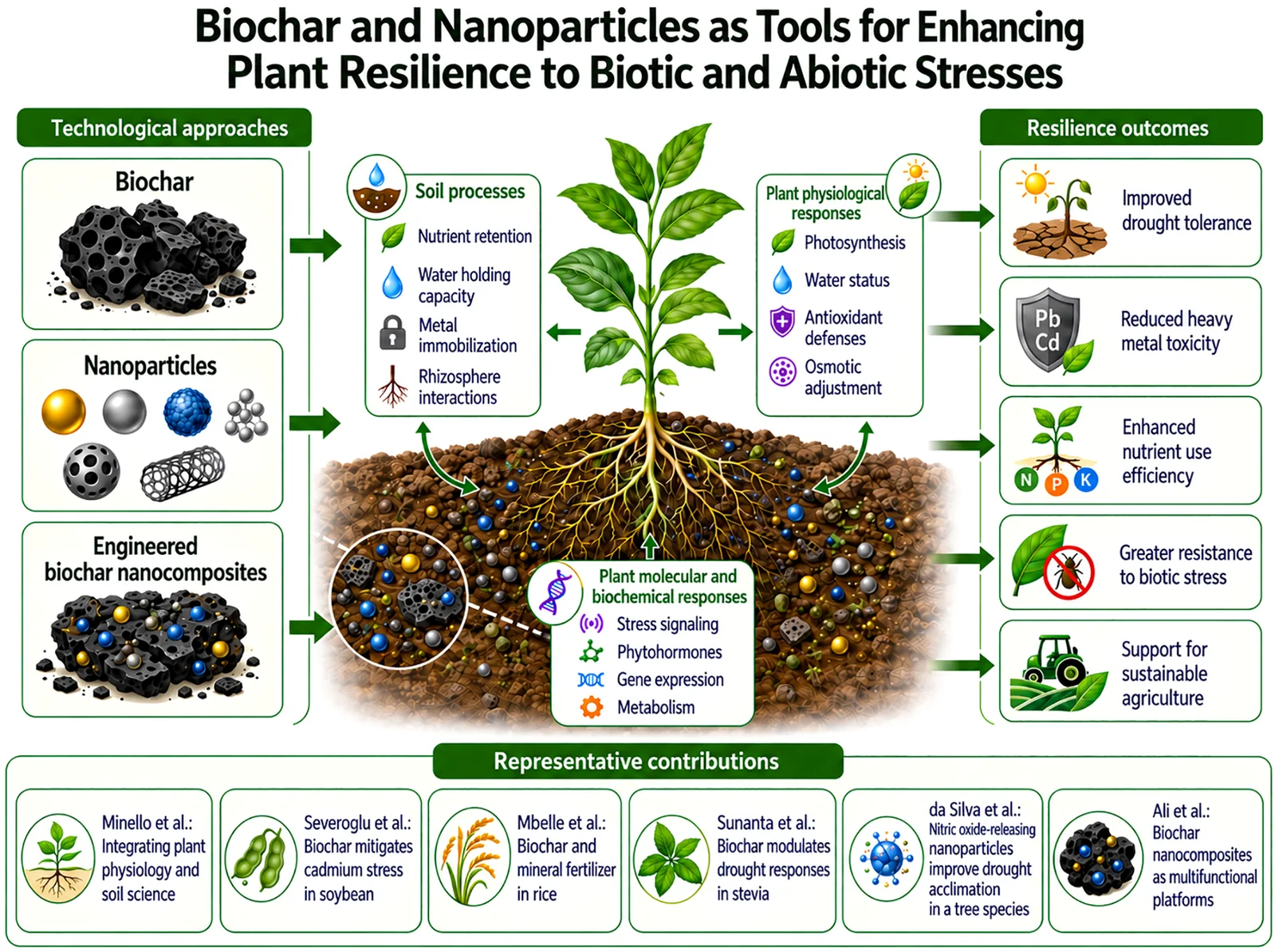
Conceptual integration of the contributions included in the Research Topic “Biochar and Nanoparticles as Tools for Enhancing Plant Resilience to Biotic and Abiotic Stresses”. Biochar, nanoparticles, and engineered biochar nanocomposites can influence interconnected processes across the soil–plant system, ranging from soil-level effects to plant physiological, biochemical, and molecular responses. These include changes in nutrient retention and availability, water relations, heavy-metal immobilization, rhizosphere interactions, photosynthesis, antioxidant defenses, osmotic adjustment, stress signaling, phytohormone regulation, gene expression, and metabolism. Together, these processes may contribute to enhanced plant resilience to biotic and abiotic stresses, improved nutrient-use efficiency, and more sustainable agricultural systems. The lower panel schematically summarizes the main conceptual contributions of the six articles included in this Research Topic.

## 2 From material effects to mechanistic understanding

This need for integration is directly addressed by Minello et al., who argue that studies involving biochar and nanomaterials should more closely connect plant physiology and soil science. The authors emphasize that assessments limited to yield or biomass are insufficient to explain how these materials improve plant performance. Instead, they advocate analyses encompassing plant-level processes, including nutrient uptake and translocation, photosynthesis, oxidative metabolism, and stress signaling, together with soil/rhizosphere processes such as nutrient dynamics, physicochemical properties, and microbial interactions.

This perspective provides an important conceptual framework for the entire Research Topic. It shifts the central question from whether a given material produces a beneficial response to how that response is generated, at which level of the soil–plant system it occurs, and under which environmental conditions it is expressed. The authors further emphasize the need to incorporate physiological, biochemical, and molecular measurements capable of distinguishing direct plant responses from indirect effects mediated by changes in soil water retention, nutrient availability, or rhizosphere functioning.

## 3 Biochar at the soil–plant interface

Three contributions illustrate the diversity of mechanisms through which biochar can influence soil–plant interactions. Severoğlu et al. investigated hazelnut-shell biochar under cadmium stress in forage soybean (*Glycine max* L. Merrill). Cadmium strongly impaired plant growth and nutrient status while increasing oxidative stress, whereas biochar mitigated several of these effects. Importantly, the response extended beyond growth traits to nutrient status, antioxidant enzyme activities, oxidative stress markers, and phytohormone regulation. These findings illustrate how biochar-mediated stress alleviation may emerge from coordinated modifications occurring both in the soil environment and within plant physiological and biochemical networks.

The study by Mbelle et al. broadens this perspective toward nutrient management under water-limited agricultural conditions. In dry direct-seeded rice (*Oryza sativa* L.), the integration of biochar with mineral fertilizer influenced plant growth and nutrient dynamics. Multivariate analysis indicated that iron and zinc, followed by total nitrogen and potassium, were among the nutrient variables most strongly associated with differences among treatments. The combined application of biochar and mineral fertilizer also promoted favorable growth responses compared with the control and fertilizer-only treatments. This study highlights an important translational aspect of biochar research: biochar may be particularly valuable when incorporated into broader nutrient-management strategies rather than being considered exclusively as an isolated agricultural input.

A more nuanced response was observed by Sunanta et al., who evaluated biochar produced from the invasive aquatic plant *Salvinia cucullata* Roxb. ex Bory in *Stevia rebaudiana* Bertoni exposed to different levels of water limitation. Biochar altered plant architecture, antioxidant-related traits, steviol glycoside accumulation, and the expression of selected biosynthesis-related genes, although improvements in biomass were not consistent across conditions. This contribution is particularly informative because it demonstrates that greater resilience should not necessarily be interpreted as a uniform increase in growth. Biochar may instead reshape metabolic allocation, antioxidant capacity, secondary metabolism, and developmental responses. In addition, conversion of invasive aquatic biomass into biochar illustrates how plant-stress mitigation strategies may also be connected with circular-economy approaches.

## 4 Nanotechnology for targeted plant stress modulation

While biochar predominantly influences the soil–plant interface, nanotechnology also offers opportunities for more targeted delivery and modulation of physiological processes. Da Silva et al. demonstrated this principle using chitosan nanoparticles loaded with *S*-nitrosoglutathione, a nitric oxide donor, in the critically endangered conifer *Araucaria angustifolia* (Bertol.) Kuntze subjected to water deficit. Nanoencapsulated *S*-nitrosoglutathione enhanced SOD and POD-associated antioxidant defense, reduced oxidative stress, promoted accumulation of soluble sugars associated with osmotic adjustment, and attenuated drought-induced growth impairment.

Beyond its relevance for drought physiology and conservation biology, this study illustrates an important advantage of nano-enabled systems: their potential to improve the delivery of unstable or short-lived signaling molecules and thereby modulate specific stress-response pathways. This represents a shift from relatively broad modification of the soil environment toward more targeted control of plant physiological and biochemical responses.

## 5 Toward integrated biochar–nanotechnology platforms

The convergence of the two main themes of this Research Topic is captured by the review of Ali et al. on engineered biochar nanocomposites. By combining the physicochemical properties of biochar with engineered nanoparticles, these systems may act across multiple levels of the soil–plant continuum. At the material and soil level, they can improve water retention and nutrient delivery, contribute to heavy-metal immobilization, and exert direct antimicrobial effects, whereas at the plant level they may promote ion and redox homeostasis and enhance defense responses, including induced systemic resistance.

Importantly, the authors move beyond a simple additive interpretation of biochar plus nanoparticles and discuss how interactions between the biochar matrix and nanomaterials may generate emergent functionalities. Their review also addresses challenges related to field performance, environmental safety, scalability, and commercialization. In this sense, engineered biochar nanocomposites represent a conceptual endpoint of the Research Topic: the transition from single-purpose amendments toward multifunctional platforms designed to address multiple constraints simultaneously, integrating soil, physiological, biochemical, and molecular mechanisms of plant resilience (**Figure 1**).

## 6 Concluding perspectives

Collectively, the contributions to this Research Topic suggest that future progress in biochar- and nanoparticle-based approaches will require not only the development of new materials, but also a better understanding and optimization of their interactions with complex biological and environmental systems. Greater emphasis is needed on realistic application rates, detailed material and soil characterization, nanoparticle transformations in the environment (e.g., dissolution, aggregation, and surface modification), plant physiological and molecular mechanisms, rhizosphere and microbiome responses, environmental fate, long-term effects, and validation under field conditions. Omics approaches and advanced analytical tools may further help connect material properties with plant responses across multiple organizational scales.

At the same time, potential benefits must be balanced against questions of ecotoxicity, persistence, scalability, cost, and regulatory feasibility. Biochar and nanoparticles should therefore not be viewed as universally beneficial inputs, but rather as context-dependent tools whose agronomic value depends on material design, environmental conditions, crop characteristics, and the specific stress being targeted. By integrating soil science, plant physiology, molecular biology, materials science, and agronomy, the studies assembled in this Research Topic contribute toward a more mechanistic and responsible framework for developing these technologies as components of resilient and sustainable agricultural systems.
